# Improving mucosal anesthesia for awake endotracheal intubation with a novel method: a prospective, assessor-blinded, randomized controlled trial

**DOI:** 10.1186/s12871-020-01210-8

**Published:** 2020-12-14

**Authors:** Chunji Han, Peng Li, Zhenggang Guo, Ying Guo, Li Sun, Gang Chen, Xiaojue Qiu, Weidong Mi, Changsheng Zhang, Lorenzo Berra

**Affiliations:** 1 Anesthesia and Operation Center, The First Medical Center of Chinese PLA General Hospital, 28th Fuxing Rd., Haidian District, Beijing, 100853 P. R. China; 2 Department of Anesthesia, The Sixth Medical Center of Chinese PLA General Hospital, Beijing, China; 3 Department of Anesthesiology, Peking University Shougang Hospital, Beijing, 100144 China; 4 Department of Gastroenterology, The First Medical Center of Chinese PLA General Hospital, Beijing, China; 5 Department of Anesthesia, Critical Care and Pain Medicine, Massachusetts General Hospital, Boston, MA USA

**Keywords:** Topical anesthesia, Awake endotracheal intubation, Dyclonine, tetracaine

## Abstract

**Background:**

Topical anesthesia is a crucial step in awake endotracheal intubation for providing favorable intubation conditions. The standard of care technique for awake intubation at our institution, which consists of oropharyngeal tetracaine spray, can result in inadequate mucosal anesthesia. Therefore, we sought to compare the effectiveness of dyclonine hydrochloride mucilage to the standard of care tetracaine in achieving anesthesia of the upper airways for awake endotracheal intubation.

**Methods:**

This is a randomized, assessor-blinded, prospective study. From Jun. 1st, 2019 to Aug. 1st, 2019, patients scheduled for either endoscopic submucosal dissection or peroral endoscopic myotomy were enrolled and randomly allocated into two groups after obtaining written informed consent: patients allocated to novel awake intubation care (Group N-AIC) received a single administration of oral dyclonine hydrochloride mucilage, whereas patients allocated to standard awake intubation care (Group S-AIC) received three oropharyngeal tetracaine sprays before transcricoid tetracaine injection before awake intubation. Mean arterial pressure (MAP), which was the primary outcome of this study, as well as heart rate (HR) were recorded throughout the procedure and compared between the two groups. Feeling of numbness, nausea, and intubation conditions after topical anesthesia were also assessed.

**Results:**

Sixty patients were enrolled and completed the study. Baseline MAP and HR were similar between the two groups. However, hemodynamic responses to intubation and gastrointestinal endoscopy, especially MAP, were significantly less elevated in Group N-AIC. The degree of numbness of the oropharyngeal mucosa after topical anesthesia did not differ between the two groups, neither did the feeling of nausea during laryngoscopy. The amount of pharyngeal secretions before intubation was less in Group N-AIC. Total intubation time was significantly shorter in Group N-AIC when compared to Group S-AIC (18.4 ± 2.86 vs. 22.3 ± 6.47, *P* < 0.05). Extubation bucking was significantly less frequent in Group N-AIC (13.3% vs. 76.7%). Patients received in Group N-AIC had a lower rate of post-extubation sore throat compared to Group S-AIC (6.7% vs. 43.3%). No adverse side effects attributable to either tetracaine or dyclonine were observed in this study.

**Conclusions:**

In awake endotracheal intubation, novel care using oral dyclonine hydrochloride mucilage can provide more favorable mucosal anesthesia and better intubation conditions compared to standard of care practice using oropharyngeal tetracaine spray.

**Trial registration:**

ChiCTR1900023151. Date of registration: May 14th, 2019.

**Supplementary Information:**

The online version contains supplementary material available at 10.1186/s12871-020-01210-8.

## Key points

**Question:** Does novel awake intubation care using oral dyclonine hydrochloride mucilage improve mucosal anesthesia for awake endotracheal intubation?

**Findings:** Novel awake intubation care using 10 ml of oral dyclonine hydrochloride mucilage is associated with fewer pharyngeal secretions, shorter intubation time, minor mean arterial pressure fluctuations, lower extubation bucking and sore throat rate perioperatively than the standard awake intubation care using three oropharyngeal tetracaine sprays (1%).

**Meaning:** In awake endotracheal intubation, novel care using oral dyclonine hydrochloride mucilage can provide more favorable intubation conditions and more stable hemodynamics than the standard of care using oropharyngeal tetracaine spray.

## Background

Awake intubation consists of placing an endotracheal tube in the trachea while a patient continues to breathe spontaneously. This technique can be utilized in many different situations to help control a potentially unstable airway. However, awake intubation can be a difficult, time-consuming maneuver for the anesthesiologist and an unpleasant experience for the patient [1]. Satisfactory execution of awake intubation needs both moderate sedation and sufficient topical anesthesia. Thus, topical anesthesia of the upper airways, including the oropharyngeal and subglottic tracheal mucosa, is a crucial element in providing adequate comfort for the patient throughout the procedure [2].

Traditionally, the standard of care at our institution for topical anesthesia of the oropharyngeal mucosa is with oropharyngeal tetracaine (0.5 ~ 1%) or lidocaine (4%) sprays, while topical anesthesia of the subglottic tracheal mucosa is provided by tetracaine (2%) transcricoid or intratracheal injection [3–6]. Tetracaine is a potent local anesthetic commonly used as the standard of care for awake intubation in many health care institutions. Application of tetracaine can effectively blunt the cough reflex and provide topical anesthesia for procedures requiring mucosal anesthesia, such as bronchoscopy and endotracheal intubation. However, the standard of care for awake intubation is potentially complicated by the undesirable properties of tetracaine sprays, including its narrow safety profile, sialogogic effects, and bitter taste [7].

Dyclonine hydrochloride, a relatively new and different chemical compound with local anesthetic properties, was initially adopted for endoscopic procedures to reduce pain, nausea, patient movements, and to lubricate the gastroscope [8]. Multiple reports have shown that dyclonine is more effective than tetracaine and lidocaine in providing adequate mucosal anesthesia [9, 10]. In daily clinical practice, the oral application of dyclonine hydrochloride mucilage does not involve as many complicated steps as tetracaine sprays do in the current standard of care regimen for awake intubation. Additionally, dyclonine can be safely used in patients with documented allergy to bupivacaine and procaine [11]. However, dyclonine is rarely utilized as a topical anesthetic for patients requiring awake endotracheal intubation. Therefore, it is worth further investigating whether dyclonine could be a potential mucosal anesthetic for use in awake intubation.

In this prospective, assessor-blinded, randomized controlled trial, the authors compared the mucosal anesthetic efficacy of oral dyclonine hydrochloride mucilage to the current standard of care, pharyngeal tetracaine sprays, for performing awake endotracheal intubation.

## Methods

This was a prospective, randomized controlled trial performed at the endoscopy center, First Medical Center of Chinese PLA General Hospital. This study was approved by the Ethical Committee of Chinese PLA General Hospital (#2019–088-01), and written informed consent was obtained from all subjects participating in the trial. The trial was registered prior to patient enrollment at the Chinese Clinical Trial Registry (ChiCTR1900023151, Principal investigator: Changsheng Zhang, Date of registration: May 14th, 2019). This manuscript adheres to the applicable CONSORT guidelines.

### Study population

From Jun. 1st, 2019 to Aug. 1st, 2019, 60 patients aged 20–65 years were enrolled in this study. Inclusion criteria included patients meeting criteria for American Society of Anesthesiology (ASA) Class I or II who were scheduled for endoscopic submucosal dissection (ESD) or peroral endoscopic myotomy (POEM) under general anesthesia. Patients were excluded if they had one or more of the following criteria: a history of asthma, known allergy to study drugs, anticipated difficult intubation, history of documented chronic organ failure, hypertension, ischemic heart disease, atrioventricular block, incomplete or partial heart blocks, application of vasoactive drugs perioperatively.

### Study procedures

Patients were randomly allocated to the standard awake intubation care group (Group S-AIC) or novel awake intubation care group (Group N-AIC) in an assessor-blinded fashion based on a computer-generated code. The anesthesiologists participating in this study had at least 5 years of experience as attending physicians at our institution. In this study, topical anesthesia administration to the upper airway was performed alone by an anesthesia nurse to ensure that the anesthesiologist, the clinical investigator, and the data analyst were all blinded to the study grouping.

After arriving in the operation room, venous access was obtained with an 18-gauge cannula placed in the left forearm. Electrocardiogram, pulse oximetry, and noninvasive blood pressure (cuff placed on the right upper arm) were continuously monitored. The standard of care for awake intubation at our institution is to perform topical anesthesia using tetracaine sprays of both the oropharyngeal mucosa and the tracheal mucosa after Bispectral index (BIS) has reached 80–85. The patient is then intubated with a video laryngoscope, video stylet, and flexible fiberoptic scope. Thus, in Group S-AIC, moderate sedation of the patient was provided with intravenous midazolam (0.03 mg/kg) and fentanyl (2 μg/kg) boluses. After adequate sedation was achieved, patients received oropharyngeal tetracaine (Chengdu Tiantaishan Pharmaceutical Co., Ltd., China) spray three times every 2 min (9 sprays and 2 intervals of 2 min in total. See supplemental video). First, the soft palate was sprayed. Then, the radix linguae were sprayed while the patient was instructed to pronounce *ha*. Finally, the epiglottis was sprayed with the guidance of delicate video laryngoscopy. The total volume of tetracaine (1%) used for oropharyngeal spray was 0.5 ml. In Group N-AIC, patients received oral administration of dyclonine hydrochloride mucilage (10 mg/10 ml, Yangtze River Pharmaceutical Co. Ltd., China) for topical anesthesia of the oropharyngeal mucosa using the same sedation index. The first 2 ml were slowly swallowed as a test-dose to rule out possible allergic reaction. After 2 min, the remaining 8 ml were administered and kept in the oropharynx for 3 min before swallowing.

The degree of oropharynx mucosal numbness was evaluated 2 min after both study procedures. After obtaining adequate pharyngeal anesthesia, needle cricothyroidotomy was performed in both groups, and 2 ml of tetracaine (2%) were injected to provide topical anesthesia of the subglottic tracheal mucosa. Three minutes later, all patients were instructed to swallow all the secretions and drug residues in the mouth and were intubated with a video laryngoscope while spontaneously breathing. The total time and number of attempted intubations were recorded.

After tube insertion, a cuff pressure between 24 and 28 cm H_2_O was maintained using an aneroid manometer to provide an adequate seal of the airway. Patients were instructed to place themselves in the left lateral position with the tube in place. General anesthesia induction was achieved by initiating target-controlled infusion (TCI) of propofol (Marsh model, target effect-site concentration of 2–4 μg/kg·min) and remifentanil (Minto model, target effect-site concentration of 3–4 ng/kg·min). The propofol and remifentanil targets were adjusted to maintain target BIS values between 40 and 60 during the entire procedure. All patients were mechanically ventilated to maintain end-tidal CO_2_ (EtCO_2_) between 32 and 36 mmHg during the surgery.

Patients’ hemodynamic parameters, mean arterial pressure (MAP) and heart rate (HR), were recorded at the following time points: 10 min after arriving in the endoscope room (T0), before the needle cricothyroidotomy (T1), immediately after intubation (T2), 5 min after intubation (T3), 3 min after left lateral positioning (T4), and immediately after extubation (T5). The degree of oropharyngeal mucosal numbness was defined as invalid, slight numbness, numbness, or significant numbness. The severity of nausea during laryngoscopy was assessed using a verbal numerical rating scale of 0–10 (0 = no feeling of nausea, 10 = severe nausea). The best view obtained by video laryngoscope in each subject was described as that which visualized the glottis or the epiglottis. The amount of secretions before intubation was classified as few, medium or heavy secretions, and the amount of suctioning required before intubation was recorded. The patient’s tolerance of endotracheal tube presence was assessed by the anesthesiologist during the patient self-positioning into left lateral decubitus as good, medium, or bad. Bucking response and presence of sore throat were recorded at extubation. The severity of sore throat at 24 h and 48 h after surgery was assessed using a verbal numerical rating scale of 0–10 (0 = no sore throat, 10 = worst sore throat imaginable).

### Statistical analysis

We anticipated enrolling 30 subjects (27 + 10% possible dropouts) in both groups. According to our pilot study, sample size calculations showed that 27 patients were needed in both groups in order to detect a difference in MAP immediately after intubation between the two groups of 7.7 mmHg (standard deviation 9.8 mmHg) with a power of 0.8 and a two-sided *p* value of less than 0.05.

The statistical analysis was conducted using Statistical Package for Social Sciences (SPSS Inc., Chicago, IL, Version 17.0 for Windows). Results are expressed as means and standard deviations, medians and ranges, or numbers and percentages. The comparison of normally distributed continuous variables between the groups was performed using *t*-test. For time-dependent changes, repeated measures analysis of variance was applied. Normality of data was checked by measures of skewness and Kolmogorov Smirnov tests of normality. Nominal categorical data between the groups were compared using the chi-squared test or Fisher’s exact test as appropriate. Ordinal categorical variables and non-normal distribution of continuous variables were compared using the Mann-Whitney *U*-test. A *p* value of less than 0.05 was considered statistically significant.

## Results

**Fig. 1:**
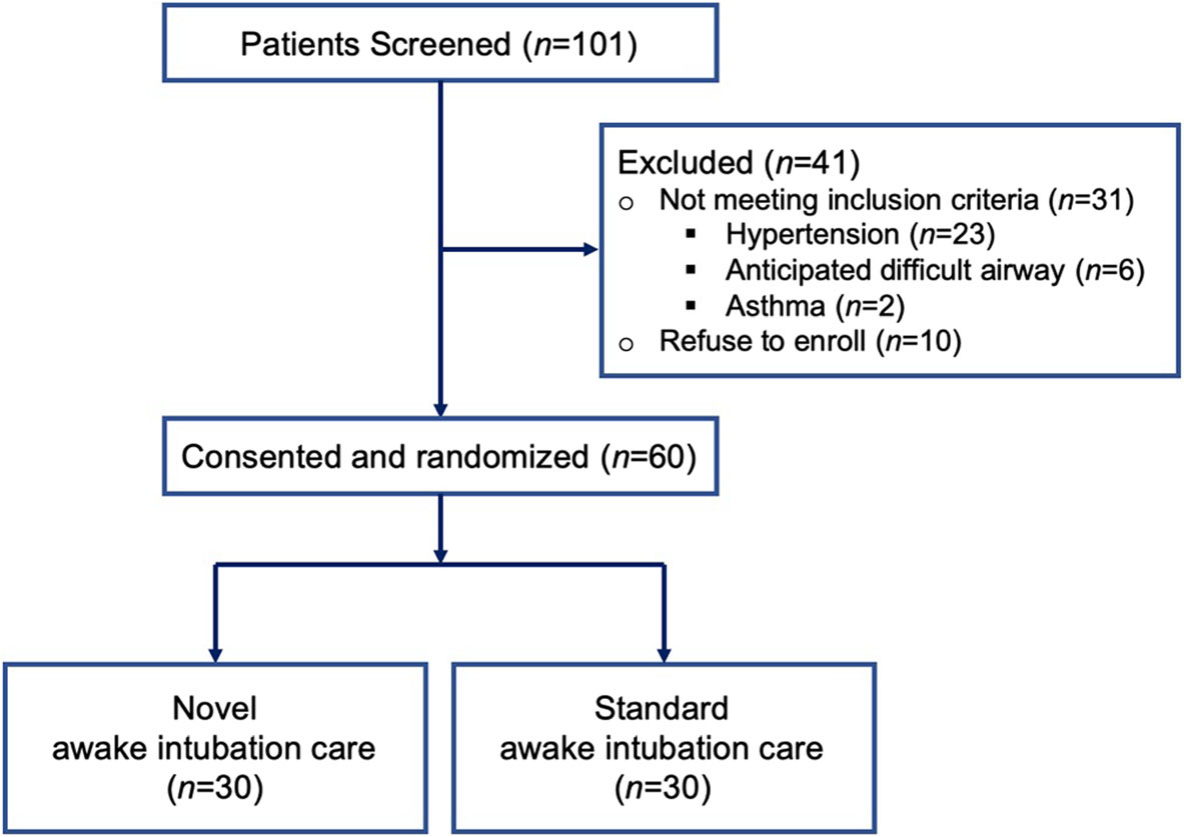
Flow chart of 101 consecutive patients scheduled for endoscopic submucosal dissection (ESD) or peroral endoscopic myotomy (POEM) under general anesthesia during the study period. After 41 patients were excluded for reasons stated above, 30 patients were randomized to novel awake intubation care, and 30 patients were randomized to standard awake intubation care

A total of 60 patients who underwent ESD or POEM were enrolled in this study. No patients were excluded from further analysis (Fig. 1). No adverse side effects of either method for awake intubation care was observed in this study. The demographic data did not differ between the two groups with regard to age and body mass index (BMI). The distribution as per sex, ASA status, and surgery type was similar in both groups, and the mean duration of surgery was comparable in both groups and statistically non-significant (Table 1).

**Table 1:** Patient characteristics

Characteristic	Group N-AIC	Group S-AIC	*P* value
Age, Median (Quartile)	55 (48–61)	54.5 (44–60)	0.51^a^
BMI, mean (SD)	24.02 (2.94)	23.78 (3.54)	0.77^b^
Sex, Male (%)	16 (53.3)	17 (56.67)	
ASA (I/II)	6/24	6/24	
Surgical procedure			
ESD (%)	25 (83.33)	25 (83.33)	
POEM (%)	5 (16.67)	5 (16.67)	
Duration of surgery (min), Median (Quartile)	63.5 (40–86)	62 (45–106)	
Fluids infused (ml), Median (Quartile)	500 (446–500)	500 (400–500)	

*BMI* Body mass index, *ASA* American Society of Anesthesiology, *ESD* Endoscopic submucosal dissection, *POEM* Peroral endoscopic myotomy, *N-AIC* Novel awake intubation care, *S-AIC* Standard awake intubation care

^a^ Two-sample *t* test. Chi-squared test

^b^ chi-squared test

### Perioperative mean arterial pressure is more stable in patients who underwent novel awake intubation care

**Fig. 2:**
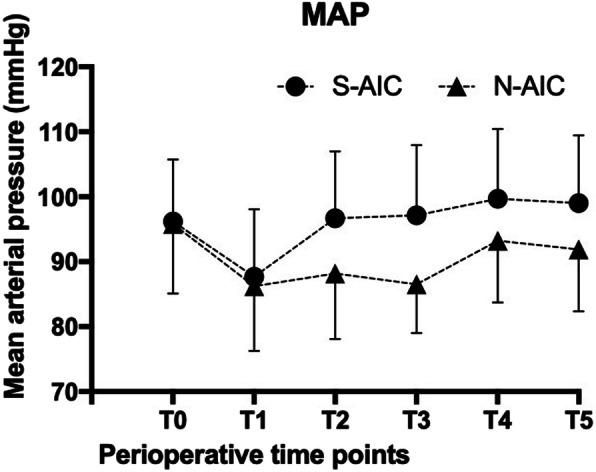
Results of mean arterial pressure (MAP) of the six perioperative time points. Error bars are +/− standard error of the mean. There were significant differences between groups at the time points of T2, T3, T4, T5 (*P* < 0.05)

**Fig. 3:**
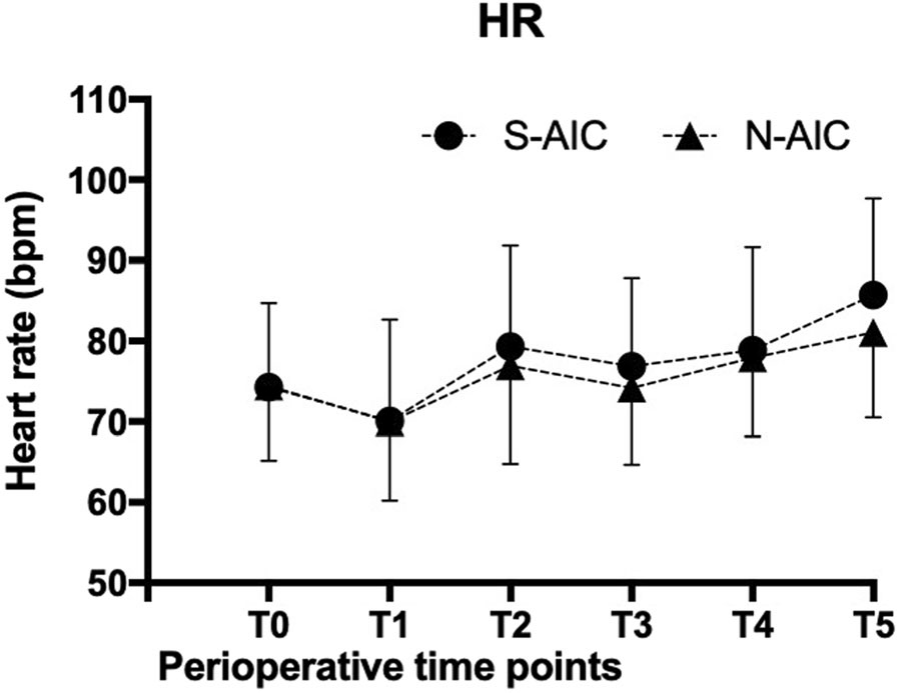
Results of heart rate (HR) of the six perioperative time points. Error bars are +/− standard error of the mean. No significant differences were found between groups

MAP and HR measurements of the six perioperative time points are summarized in Figs. 2 and 3. The baseline (T0 and T1) MAP and HR were comparable between the two groups. However, there was an overall statistically significant differences among the two groups regarding MAP immediately after intubation (T2) and subsequent time points T3 and T4 (*P* < 0.0083, Bonferroni corrected significance level). The novel awake intubation care using dyclonine was found to significantly reduce MAP at intubation and left lateral positioning when compared to standard awake intubation care. However, although the mean HR in the N-AIC group was slightly lower at T2, T3, T4, and T5 as compared with the S-AIC group, the difference was not statistically significant (*P* = 0.124) (Table 2).

**Table 2:** Comparison of MAP and HR between the study groups at various time points

Time points	Parameter	Group N-AIC		Group S-AIC		*P* Value^a^
		Mean	SD	Mean	SD	
T0	MAP (mmHg)	95.83	10.74	96.17	9.57	0.899
	HR (bpm)	74.43	9.25	74.27	10.46	0.948
T1	MAP (mmHg)	86.23	9.98	87.70	10.39	0.579
	HR (bpm)	69.97	9.78	70.07	12.62	0.630
T2	MAP (mmHg)	88.17	10.11	96.67	10.32	0.002*
	HR (bpm)	76.90	12.16	79.33	12.49	0.448
T3	MAP (mmHg)	86.53	7.49	97.13	10.79	0.000*
	HR (bpm)	74.17	9.51	76.87	10.97	0.312
T4	MAP (mmHg)	93.23	9.50	99.70	10.75	0.000*
	HR (bpm)	77.90	9.77	78.87	12.757	0.743
T5	MAP (mmHg)	91.90	9.54	99.03	10.42	0.008*
	HR (bpm)	81.10	10.58	85.73	11.942	0.120

*MAP* Mean arterial pressure, *HR* Heart rate, *SD* Standard deviation

* *P* < 0.05 considered statistically significant

^a^ Repeated measurement analysis of variance

### Patients who underwent novel awake intubation care had fewer secretions in the oropharynx and shorter intubation duration

**Fig. 4:**
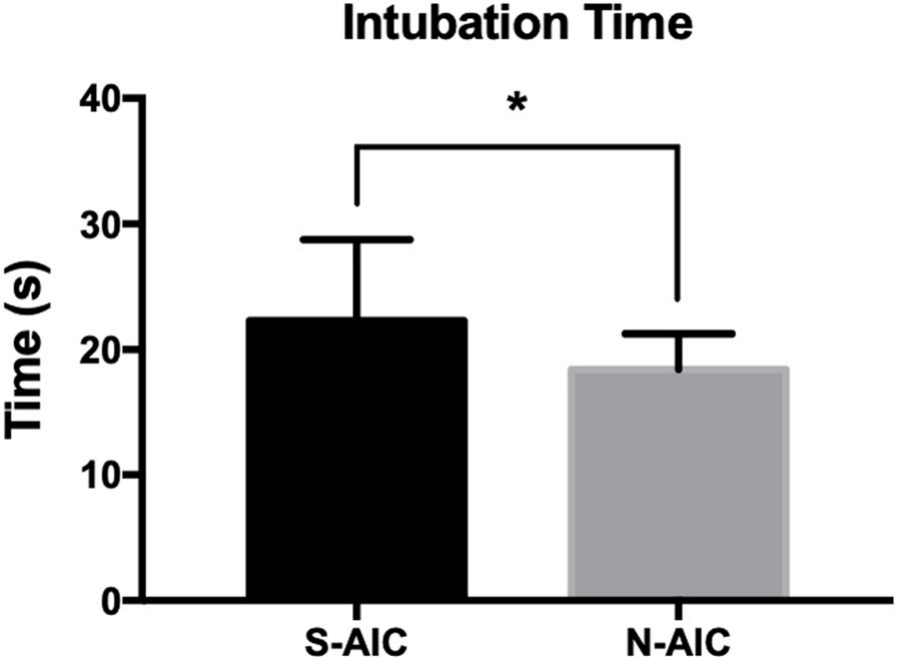
Intubation time between groups. Each vertical bar represents the mean ± standard error (*n* = 30 in each group). The total time of intubation was significantly shorter in the N-AIC group (*P < 0.05)

The amount of secretions in patients’ oropharynx before intubation was less in the N-AIC group compared to the S-AIC group (*P* = 0.01). Favorable intubation conditions (visualized glottis) were reported in 30 patients in the N-AIC group and 27 patients in the S-AIC group (*P* = 0.237). No patients in the N-AIC group required suctioning before intubation, and only three patients in the S-AIC group required upper airway suctioning before intubation (*P* = 0.237). The total time of intubation was significantly shorter in the N-AIC group (18.4 ± 2.86 vs. 22.3 ± 6.47, *P* < 0.05, Fig. 4). All the patients in the N-AIC group were successfully intubated at the first attempt, while only one patient in the S-AIC group required two attempts. The endotracheal tube was well tolerated in lateral decubitus in most patients, except for two patients in the S-AIC group whose tolerance was reported as “*medium*”.

### Novel awake intubation care did not improve patients’ subjective sensation but reduced extubation bucking and sore throat at 24 h after extubation

The degree of numbness of the oropharyngeal mucosa after topical anesthesia and the feeling of nausea during laryngoscopy were not different between the two groups (*P* = 0.546 and *P* = 0.317, respectively). However, only 13.3% of patients who received N-AIC had bucking at extubation, compared to 76.7% with S-AIC (*P* < 0.001). Patients in the N-AIC had a much lower rate of extubation sore throat than the S-AIC group (6.7% vs. 43.3%, *P* < 0.001). At 24 h after surgery, the severity of sore throat was significantly lower in the N-AIC group than the S-AIC group (0[0–1] vs. 3[0–4], *P* = 0.001). However, this difference in throat soreness between the two groups did not achieve statistical significance at 48 h after surgery (0[0–0] vs. 0[0–0], *P* = 0.31).

## Discussion

In this prospective, randomized, controlled trial, we compared the effect of two different types of topical anesthesia for awake intubation care, using oral dyclonine hydrochloride mucilage (10 mg/10 ml) or tetracaine spray (1%, 0.5 ml). We found that awake intubation care using oral dyclonine hydrochloride mucilage provides more favorable intubation conditions and better hemodynamics during the perioperative periods than standard awake intubation care.

The reason why awake intubation is a routine method for patients who undergo general anesthesia at our endoscopy center is because it is a relatively safer method for airway management, which could decrease potential aspiration risks and free from muscle relaxants perioperatively. Thus, satisfactory methods for awake intubation are always taken into account. Topical anesthesia of the oropharyngeal mucosa is of crucial importance before awake intubation, and can provide the patient with a relatively comfortable feeling during the laryngoscopic examination. Adequate topical anesthesia of the oropharyngeal mucosa could also result in better cooperation from the patient. Induction of amnesic sedation followed by awake intubation is a common technique at our institution. Given the rapid absorption of tetracaine in the pharynx, tetracaine spray is routinely used to provide topical anesthesia for fiberoptic tracheal intubation and other procedures requiring mucosal anesthesia. In addition, tetracaine frequently provides topical anesthesia for gastrointestinal and ocular procedures. It is reported that the maximum effective concentration of tetracaine is 1%, with a latent period of 1.1 min and a duration of 55.5 min [12].

However, in our experience, topical tetracaine is far from being the ideal mucosal anesthetic due to the following two reasons. First, the bitter taste and feeling of nausea after oropharyngeal application cause patients to regurgitate or swallow the drug, which significantly reduces the amount of drug acting on the mucosa. Additionally, anesthesiologists who are not experienced in administering topical anesthesia of the oropharyngeal mucosa often leave some parts of the mucosa un-anesthetized, and thus still sensitive to stimulation. Stevens et al. tested the effects of tetracaine inhalation before intubation. He found that a small dose of nebulized tetracaine may completely coat mucosal surfaces and significantly attenuates the hemodynamic response to tracheal intubation [13]. Therefore, complete coating of mucosal surfaces is a crucial factor of successful topical anesthesia. This may explain why the effects of tetracaine spray used in the S-AIC group is not as good as dyclonine hydrochloride mucilage used in N-AIC in attenuating the hemodynamic response of intubation.

Dyclonine hydrochloride mucilage is a topical anesthetic that reversibly binds to activated sodium channels on the neuronal membrane, thereby decreasing the neuronal membrane’s permeability to sodium ions, leading to an increased threshold for excitation [14]. It very effectively produces topical anesthesia and lubricates the mucosal surfaces for gastrointestinal endoscopy and endotracheal intubation [15, 16]. When applied to mucus membranes, the onset of topical anesthesia is 2-10 min and lasts for 20–30 min [12]. Beginning in 1983, the safety and effectiveness of dyclonine were recognized as superior to lidocaine and tetracaine for awake intubation [17]. In 1997, Bacon et al. reported the use of oral and nebulized dyclonine for topical anesthesia of the airway to facilitate awake intubation in a patient with a stated allergy to bupivacaine and procaine [11]. In our study, we found that dyclonine hydrochloride mucilage may have a more prolonged effect than reported because the incidence of extubation bucking and sore throat was significantly lower in the N-AIC group, factors that contributed in providing a better anesthesia and intubation experience to patients.

In this study, we did not find any difference in the subjective sensation of topical anesthesia between the two kinds of awake intubation care, which demonstrate the similar anesthetic effects of both drugs after proper administration. However, patients who received N-AIC had a significantly better hemodynamic profile during and after the procedure, suggesting that dyclonine mucilage significantly attenuates hemodynamic distress induced by laryngoscopy and endotracheal intubation, especially if compared to tetracaine.

Notably, the duration of the intubation process was significantly shorter in patients who received novel awake intubation care. The defoaming effect of dyclonine hydrochloride mucilage, which eliminate the mucous bubbles in the oral-pharyngeal cavity, leads to a better view and fewer visible secretions during the videolaryngoscopic examination [18]. Thus, dyclonine mucilage administration not only provides better mucosal anesthesia (i.e., better attenuates hemodynamic response of laryngoscopy and intubation, is an efficacious anesthetic effect, and leads to excellent patient cooperation), but can also provide better intubation conditions (i.e., fewer visually obstructive secretions and faster intubation).

However, the limitations of our study must be acknowledged. The subjective assessments of the intubation condition by anesthesiologists, and the subjective sensation of the numbness of oropharynx mucosa, nausea and sore throat reported by the patients may lead to subjective bias, which may be the underlying reason for lack of statistically significant differences in some of these parameters. The sedation level prior to intubation was not recorded, which may lead to a difference in subjective sensation during and after topical anesthesia among the patients. Future studies are expected to reveal the effects of dyclonine as a nasal mucosal topical anesthetic during nasotracheal intubation and could provide more comprehensive information for its clinical application.

## Conclusion

In awake endotracheal intubation, novel care using oral dyclonine hydrochloride mucilage can provide more favorable mucosal anesthesia and better intubation conditions than standard of care using oropharyngeal tetracaine sprays.

## Supplementary Information

**Additional file 1: Supplementary Video.** The video is a demonstration of the standard awake intubation care in the First medical center of Chinese PLA General Hospital. First, a certain sedation basis was provided by intravenous midazolam (0.03 mg/kg) and fentanyl (2 μg/kg) boluses. Then, the patient received oropharyngeal tetracaine spray (1%) every 2 min for three times. 1. The soft palate was sprayed. 2. The radix linguae was sprayed while the patient was instructed to pronounce *ha*. 3. The epiglottis was sprayed after exposure through delicate video laryngoscopy. Two minutes later, needle cricothyroidotomy was performed, and 2 ml of tetracaine (2%) were injected to provide topical anesthesia of subglottic tracheal mucosa. Three minutes later, the patient was instructed to swallow all the secretions and drug residues in the mouth and intubated with a video laryngoscope. After successful intubation, the patient was instructed to place herself to the left lateral position with the tube in place before the surgery.

## Data Availability

The datasets used and/or analyzed during the current study are available from the corresponding author on reasonable request.

## Abbreviations

S-AIC: Standard awake intubation care
N-AIC: Novel awake intubation care
ASA: American Society of Anesthesiology
ESD: Endoscopic submucosal dissection
POEM: Peroral endoscopic myotomy
BIS: Bispectral index
TCI: Target-controlled infusion
EtCO_2_: End-tidal CO_2_
MAP: Mean arterial pressure
HR: Heart rate
